# BK Polyomavirus Genotypes in Two Patients after Hematopoietic Cell Transplant

**DOI:** 10.1128/MRA.01122-20

**Published:** 2021-01-14

**Authors:** Elizabeth A. Odegard, Heidi L. Meeds, Steven B. Kleiboeker, Assem Ziady, Anthony Sabulski, Sonata Jodele, Stella M. Davies, Benjamin L. Laskin, Jason T. Blackard

**Affiliations:** a Division of Digestive Diseases, University of Cincinnati College of Medicine, Cincinnati, Ohio, USA; b Viracor-Eurofins Laboratories, Lee’s Summit, Missouri, USA; c Divsion of Pulmonary Medicine, Cincinnati Children’s Hospital Medical Center, Cincinnati, Ohio, USA; d Division of Bone Marrow Transplantation and Immune Deficiency, Cincinnati Children’s Hospital Medical Center, Cincinnati, Ohio, USA; e Division of Nephrology, Children’s Hospital of Philadelphia, Philadelphia, Pennsylvania, USA; DOE Joint Genome Institute

## Abstract

BK polyomavirus (BKPyV) infection can lead to nephropathy and hemorrhagic cystitis (HC). We evaluated BKPyV genotypes in two individuals after hematopoietic cell transplant (HCT). The first case developed HC and was infected with genotype Ib-2, while the second did not develop HC and was infected with genotype Ia.

## ANNOUNCEMENT

Human BK polyomavirus (BKPyV) is a circular, double-stranded DNA virus of the *Polyomaviridae* family (genus *Betapolyomavirus*) that infects up to 90% of the general population by 10 years of age (1). While BKPyV in immunocompetent individuals is rarely associated with clinical disease, during periods of immunosuppression, primary infection or reactivation of latent virus can lead to kidney (BKPyV-associated nephropathy) and/or bladder (hemorrhagic cystitis [HC] and ureteral stenosis) injury (2, 3). There are no antiviral therapies approved for the treatment of BKPyV infection. Thus, a comprehensive understanding of viral diversity is essential for the development of future therapeutic approaches (4).

We performed BKPyV genotype analysis in two patients after hematopoietic cell transplant (HCT). One developed clinical disease, while the other did not. Subjects were enrolled in a prospective observational cohort and specimen repository after their HCT (5). Briefly, BKPyV viruria and viremia were quantified in urine and blood samples from children and young adults undergoing HCT at Cincinnati Children’s Hospital Medical Center (CCHMC) between April 2013 and May 2018. Nucleic acid testing for BKPyV was performed on all plasma and urine samples at Viracor-Eurofins with a lower limit of quantification of 39 copies per milliliter. The CCHMC Institutional Review Board approved the study, and all patients or their parents/guardians provided written informed consent/assent. DNA was extracted using the Qiagen QIAamp UltraSens virus kit with centrifugation optimized for urine. The eluted DNA was digested with BamHI at 37°C for 60 min. The whole BKPyV circular genome was amplified using the Qiagen long-range PCR kit with BK1731F and BK1739R primers and reaction conditions (6). Additionally, a nested PCR was performed to amplify a 1.5-kb region of the VP1 (7). PCR products were run on an agarose gel and purified with the QIAquick gel extraction kit. Next-generation sequencing (NGS) library preparation was performed using the NEB NEBNext Ultra II FS DNA library prep kit and sequenced with the setting of SR 1 × 51 bp on an Illumina HiSeq 1000 sequencer. PCR products were prepared separately, and the resulting reads were combined for samples from the same individuals. All tools were run with default parameters unless otherwise specified. The reads generated were run through FastQC; no reads were characterized as poor quality. Case 1 yielded a total of 2,268,367 reads with an average depth of 22,502×, while case 2 yielded 540,314 reads with an average depth of 5,360×. The reads were mapped to the BKPyV reference genome sequence Dunlop (GenBank accession number V01108) within UGENE version 36.0, and the resulting consensus sequence was generated from each individual (8). For case 1, the genome sequence was 4,275 nucleotides long with a G+C content of 38%. For case 2, the genome sequence was 4,657 nucleotides long with a G+C content of 39%. The BKPyV genotype was initially assigned using the BKTyper tool (9). Phylogenetic inference was then performed using the Bayesian Markov chain Monte Carlo (MCMC) method in the Bayesian Evolutionary Analysis by Sampling Trees (BEAST) program version 1.10.1 (10) with a chain length of 500,000,000. After a 10% burn-in using Tree Annotator version 1.10.1, the maximum clade credibility tree was visualized in FigTree version 1.4.4.

Case 1 is a male who underwent allogeneic HCT at age 15 years for chronic granulomatous disorder. BKPyV viremia was first noted on day +11 after HCT, but he remained asymptomatic until HC developed on day +109. The urine BKPyV copy number at the time of symptom onset was 1.7 × 108 copies per milliliter, and the plasma BKPyV was 140,302 copies per milliliter.

Case 2 is a female who underwent myeloablative allogeneic HCT at age 21 years for acute myelogenous leukemia. BKPyV was detected on day +28 with viremia of 900 copies per milliliter and viruria of 8.0 × 109 copies per milliliter. BKPyV viremia was also detected at month 2 (2,100 copies) and month 4 (2,900 copies). BKPyV viruria persisted during the first 4 months after transplant, peaking at month 1 and decreasing to 771,000 copies per milliliter by month 4.

**FIG 1:**
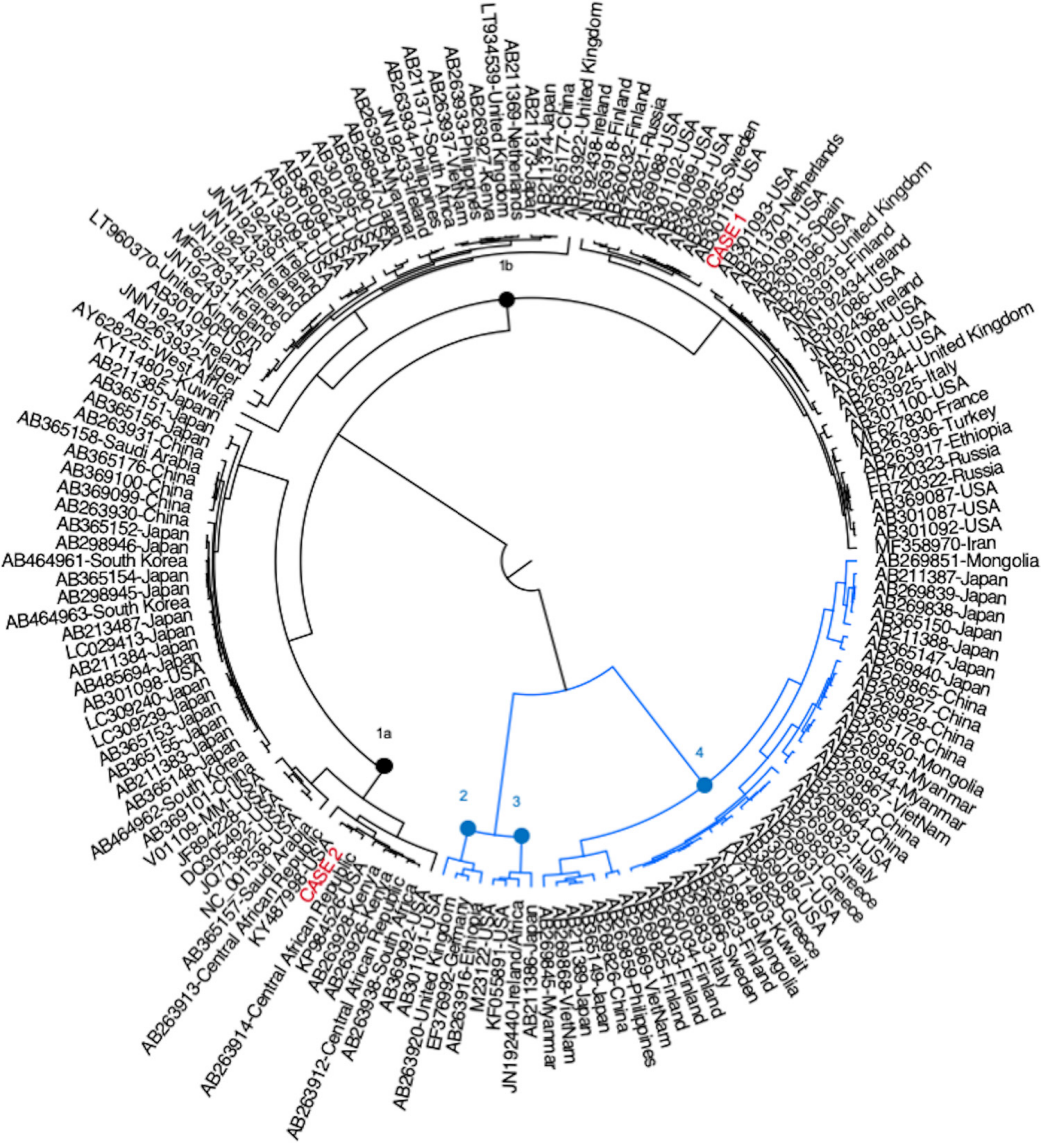
Representative BKPyV genome sequences were downloaded from GenBank, and phylogenetic inference was performed in BEAST version 1.10.1 (10). Sequences for cases 1 and 2 are highlighted in red, while GenBank references are labeled by their accession number and country of origin. Genotypes 2, 3, and 4 are shown in blue. The nodes separating all genotypes are highlighted with closed circles.

Comparison to other BKPyV genome sequences confirmed that case 1 clustered with genotype Ib-2 references, while case 2 clustered with genotype Ia references (Fig. 1). Further understanding of viral pathogenesis and BKPyV diversity may define the mechanisms of kidney and bladder injury and inform the development of targeted therapeutic approaches for these high-risk patients.

### Data availability.

The raw sequence reads are available under BioProject accession number PRJNA670723 with reads available in the SRA under accession numbers SRX9346189 (case 2) and SRX9346190 (case 1). Consensus BKPyV genome sequences are available under GenBank accession numbers MW023596 (case 2) and MW023597 (case 1).
